# A Case of Epileptic Convulsions Cured by the Internal Administration of Chloroform

**Published:** 1853

**Authors:** H. Bowe


*A Case of Epileptic Convulsions cured by the Internal Administration of Chloroform,*—By H. Bowe, Esq.

The following case possesses several points of interest:—

Thomas Eason, aged one year and seven months, was placed under my care on the 7th of April, 1853, suffering from epileptic fits.

The previous history of the case, as obtained from the mother, is as follows:—When the child was five months old, she was awoke early in the morning by a scream, and then found him in strong convulsions, the face turgid, the mouth foaming, and the hands forcibly clenched. Being much alarmed, she took him to a medical man, who attended him for some time, but gave her no hope of his ultimate recovery, as he feared there was organic disease of the brain. He was subsequently attended by two other medical men; the fits at this period occurring from three to six times in the course of the day. The remedies prescribed seemed to affect neither their force nor frequency. As the mother could not afford to pay for medical advice, the child for some time continued in about the same state, without any means being taken for his relief. At the time he first came under my care, (April 7th) he frequently had seven or eight well-marked fits during the day. His appearance was very peculiar; the head being either constantly rolled from side to side, or thrown backwards, his face had a vacant and almost idiotic expression. On looking into the mouth, I found the gums covering the upper incisor teeth, tense and swollen; they were freely lanced, and an aperient powder given. The child to be put into a warm bath.

10th.—The fits had increased in frequency, the child was no better in any respect. As the gums were still rather tenser, they were again freely incised, another warm bath ordered, as well as another dose of castor oil. As the child was continually rubbing his nose, I subsequently ordered him a powder of calomel and jalap, thinking, perhaps, he had worms. The bowels were freely relieved, but no worms were detected.

From this time up to April 20th, the fits increased rapidly; as many as eighteen occurred in one dav, following each other in quick succession. In the short intervals between them, he lay in a semi-comatose condition; for three days he took no nourishment whatever, and was evidently fast sinking from exhaustion. Seeing that everything done had failed to give even the slightest relief, I determined to give chloroform a trial.

I commenced giving it internally, in five minim doses, suspended in mucilage, directing the mother, after every fit, to give a dose of the medicine. On visiting the child the following morning, the 21st, I found that he had slept almost continuously since taking the first dose, and that there had not been any fit. He was quite sensible when awakened to take his food or medicine, of which three doses had been given in the course of the previous day.

I directed the mother to continue the medicine three times daily, and frequently to give small quantities of beef-tea and arrowroot. From this date the child improved rapidly up to May 6th, on which day he was very fretful, and evidently suffering from the irritation caused by the teeth, which had nearly penetrated the gums. The dose of chloroform was increased to seven minims, and a purgative powder to be given twice a-week, after which he was much relieved. I ceased to attend him on May 20th. He was then perfectly well, never having had a return of the fits since the first dose of chloroform was administered.

July 18th.—I have this day seen the child; he continues in perfect health; has cut three teeth, and has not had any return of fits.

I am not aware that chloroform has hitherto been given internally in similar cases to the present. Of course, very little can be argued from a solitary case; but, perhaps, experience will prove that in convulsive affections, especially those of children, we have in chloroform a powerful remedial agent.

*Medical Times and Gazette*, Sept. 24, 1853.

